# The cardiac sympathetic co-transmitter neuropeptide Y is pro-arrhythmic following ST-elevation myocardial infarction despite beta-blockade

**DOI:** 10.1093/eurheartj/ehz852

**Published:** 2019-12-13

**Authors:** Manish Kalla, Guoliang Hao, Nidi Tapoulal, Jakub Tomek, Kun Liu, Lavinia Woodward, ‘Oxford Acute Myocardial Infarction (OxAMI) Study’, Erica Dall’Armellina, Adrian P Banning, Robin P Choudhury, Stefan Neubauer, Rajesh K Kharbanda, Keith M Channon, Olujimi A Ajijola, Kalyanam Shivkumar, David J Paterson, Neil Herring

**Affiliations:** 1 Department of Physiology, Anatomy and Genetics, Burdon Sanderson Cardiac Science Centre, University of Oxford, Parks Road, Oxford OX13PT, UK; 2 Department of Cardiovascular Medicine, John Radcliffe Hospital, University of Oxford, Oxford OX3 9DU, UK; 3 Radcliffe Department of Medicine, Acute Vascular Imaging Centre, University of Oxford, Oxford OX3 9DU, UK; 4 UCLA Cardiac Arrhythmia Center and Neurocardiology Research Center, Los Angeles, CA, USA

**Keywords:** Neuropeptide Y, Myocardial infarction, Percutaneous coronary intervention, Ventricular tachycardia, Ventricular fibrillation

## Abstract

**Aims:**

ST-elevation myocardial infarction is associated with high levels of cardiac sympathetic drive and release of the co-transmitter neuropeptide Y (NPY). We hypothesized that despite beta-blockade, NPY promotes arrhythmogenesis via ventricular myocyte receptors.

**Methods and results:**

In 78 patients treated with primary percutaneous coronary intervention, sustained ventricular tachycardia (VT) or fibrillation (VF) occurred in 6 (7.7%) within 48 h. These patients had significantly (*P* < 0.05) higher venous NPY levels despite the absence of classical risk factors including late presentation, larger infarct size, and beta-blocker usage. Receiver operating curve identified an NPY threshold of 27.3 pg/mL with a sensitivity of 0.83 and a specificity of 0.71. RT-qPCR demonstrated the presence of NPY mRNA in both human and rat stellate ganglia. In the isolated Langendorff perfused rat heart, prolonged (10 Hz, 2 min) stimulation of the stellate ganglia caused significant NPY release. Despite maximal beta-blockade with metoprolol (10 μmol/L), optical mapping of ventricular voltage and calcium (using RH237 and Rhod2) demonstrated an increase in magnitude and shortening in duration of the calcium transient and a significant lowering of ventricular fibrillation threshold. These effects were prevented by the Y_1_ receptor antagonist BIBO3304 (1 μmol/L). Neuropeptide Y (250 nmol/L) significantly increased the incidence of VT/VF (60% vs. 10%) during experimental ST-elevation ischaemia and reperfusion compared to control, and this could also be prevented by BIBO3304.

**Conclusions:**

The co-transmitter NPY is released during sympathetic stimulation and acts as a novel arrhythmic trigger. Drugs inhibiting the Y_1_ receptor work synergistically with beta-blockade as a new anti-arrhythmic therapy.

**See page 2180 for the editorial comment on this article (doi: 10.1093/eurheartj/ehz950)**

## Introduction

Acute myocardial infarction (MI) is associated with high levels of cardiac sympathetic drive^1^ which is a strong prognostic indicator for adverse outcomes and can trigger life-threatening ventricular arrhythmias.^2^^,^^3^ Despite significant improvements in mortality with emergency reperfusion and modern pharmacological treatment, large ST-elevation myocardial infarction (STEMI) is complicated by ventricular arrhythmias in up to 10% of cases.^4^ Indeed, the only primary prevention anti-arrhythmic drugs that reduce mortality following acute MI are beta-blockers.^5^ Prolonged cardiac sympathetic stimulation^6^ or experimentally induced MI^7^ in animal models also causes the release of non-catecholaminergic co-transmitters such as neuropeptide Y (NPY). We have shown that both local cardiac and circulating levels of the NPY are significantly elevated in patients undergoing primary percutaneous coronary intervention (PPCI) following STEMI^8^ and remain high over at least 48 h following the event.^9^

Interestingly, many species including rat and human, are known to express NPY receptors on cardiomyocytes,^10^ and NPY can influence calcium handling and electrophysiology. For example, in isolated rat ventricular myocytes, NPY increases the size of intracellular calcium transients and promotes diastolic calcium release events from the sarcoplasmic reticulum,^11^ which are known to cause delayed afterdepolarizations via the electrogenic Na^+^/Ca^2+^ exchange (NCX) and trigger arrhythmic events.^2^ In addition, patch-clamp studies of isolated ventricular myocytes have shown that NPY can increase the transient outward potassium current (*I*_to_),^12^ and reduce the L-type calcium current (*I*_CaL_).^13^ This may shorten action potential duration, reduce refractory period, and help sustain re-entrant arrhythmias.

We therefore hypothesized that despite maximal beta-blockade, NPY released during sympathetic stimulation can promote ventricular arrhythmias in a novel Langendorff perfused rat heart preparation with intact stellate ganglia and sympathetic innervation, and explore the underlying mechanisms. We also sought to ascertain whether patients undergoing PPCI for STEMI with high plasma NPY levels were therefore more likely to experience ventricular arrhythmias. To further investigate possible causation, we tested whether NPY promotes arrhythmias during experimental ST-elevation ischaemia/reperfusion in the rat and explored the receptor pathways involved to assess whether these may be applicable for human pharmacological intervention.

## Methods

See Supplementary material online for expanded methods. Patients with left coronary artery STEMI were recruited as part of the Oxford Acute Myocardial Infarction (OxAMI) study, which was approved by local ethics committee (REC: 10/H0408/24). All participants gave written informed consent. Stellate ganglia were collected from organ donors at the time of organ procurement as approved by the UCLA IRB and written informed consent was provided by the patient or appropriate designee. These studies comply with the Declaration of Helsinki. Animal use complied with the University of Oxford local ethical guidelines and was in accordance with the Guide for the Care and Use of Laboratory Animals published by the US National Institutes of Health (NIH Publication No. 85-23, revised 2011) and the Animals (Scientific Procedures) Act 1986 (UK). Experiments were performed under British Home Office Project License PPL 30/3131.

### Statistical analysis

Data are presented as mean ± standard deviation, or as median [interquartile range] if data did not pass a normality test (Shapiro–Wilk). A paired *t*-test was used to compare grouped data with two measures, whilst a one-way analysis of variance (ANOVA) was applied to grouped data with more than two measures, with *post hoc* analysis to determine significance (Neuman–Keuls). An unpaired *t*-test assuming unequal variance was used to determine significance between independent groups. Non-parametric data from two independent groups were compared using a Mann–Whitney *U* test and for more than two groups using a Kruskal–Wallis one-way ANOVA. Discrete data were analysed using χ^2^ or Fisher’s exact test in contingency tables. All significance tests are two-tailed and significance accepted at *P* < 0.05.

## Results

### Neuropeptide Y levels and ventricular arrhythmias in ST-elevation myocardial infarction patients following primary percutaneous coronary intervention

A total of 78 patients with acute left coronary artery STEMI (presenting throughout the 24 h cycle of clinical activity) were recruited and underwent peripheral venous blood sampling at the time of PPCI. Overall patients were 63 ± 12.4 years old and 61/78 (78.2%) were male. The mean pain to balloon time was 218 ± 159 min. Peripheral venous NPY concentration was 19.0 [11.7–31.7] pg/mL. For comparison, the venous NPY level measured during elective coronary angiography in 12 patients of a similar age and sex, with similar cardiovascular risk factors but with unobstructed coronary arteries was 7.8 [6.5–12.2] pg/mL (see *Table 1*).

**Table 1:** Venous NPY concentrations and patient details according to clinical diagnosis

	Normal coronary arteries (*n* = 12)	STEMI (*n* = 78)	*P*-value
Age	64.7 ± 12.1	63.0 ± 12.4	0.68
Males	7/12 (58.3%)	61/78 (78.2%)	0.16
Cardiovascular risk factors			
Hypertension	9/12 (75%)	36/78 (46.2%)	0.12
Hyperlipidaemia	7/12 (58.3%)	32/78 (41.0%)	0.42
Diabetes mellitus	2/12 (16.7%)	10/78 (12.8%)	1.00
Current/Ex-smoker	7/12 (58.3%)	61/78 (78.2%)	0.16
Family history	6/12 (50.0%)	26/78 (33.3%)	0.33
Previous MI	3/12 (25%)	7/78 (9.0%)	0.13
Venous NPY concentration (pg/mL)	7.8 [6.5–12.2]	19.0 [11.7–31.7]	<0.001

Values are mean ± standard deviation, *n* (%), median [interquartile range].

MI, myocardial infarction; STEMI, ST-elevation myocardial infarction.

Sustained VT/VF was observed in 7.7% (6/78) of the STEMI patients. The individual characteristics of these patients in terms of pain to balloon time, arrhythmia timing, and termination are detailed in Supplementary material online, *Table S1*. Known clinical risk factors for ischaemia reperfusion arrhythmias following STEMI include cardiogenic shock, late presentation, larger infarct size, and prior beta-blocker usage.^14^ No patients in cardiogenic shock were included in our study, and patients with VT/VF in our cohort had a significantly faster pain to balloon time compared to the rest of the cohort, and a similar proportion had previous MIs and prior beta-blocker use (as shown in *Table 2*). Ejection fraction, oedema, and late gadolinium enhancement (LGE) on cardiac magnetic resonance imaging (MRI) at 48 h were also similar. However, patients with VT/VF had significantly higher venous plasma NPY levels compared to the rest of the study group (31.9 [27.8–47.7] vs. 17.8 [10.6–30.2] pg/mL).

**Table 2:** Clinical characteristics in patients with and without sustained VT/VF following STEMI

STEMI patients	No VT/VF (*n* = 72)	VT/VF (*n* = 6)	*P*-value
Age	63.0 ± 12.7	63.7 ± 14.5	0.91
Males	56/72 (77.8%)	5/6 (83.3%)	1.00
Cardiovascular risk factors			
Hypertension	33/72 (45.8%)	3/6 (50.0%)	1.00
Hyperlipidaemia	5/72 (6.9%)	1/6 (16.7%)	0.39
Diabetes mellitus	10/72 (13.9%)	0/6 (0.0%)	0.59
Current/Ex-smoker	58/72 (80.6%)	3/6 (50.0%)	0.11
Family history	23/72 (31.9%)	3/6 (50.0%)	0.66
Previous MI	5/72 (6.9%)	2/6 (33.3%)	0.09
Medications on admission			
Beta-blockers	11/72 (15.3%)	1/6 (16.7%)	1.00
ACE inhibitor/ATR antagonist	17/72 (23.6%)	1/6 (16.7%)	1.00
Statin	17/72 (23.6%)	1/6 (16.7%)	1.00
BP and heart rate at presentation			
Systolic BP (mmHg)	134.6 ± 25.5	135.2 ± 22.8	0.94
Diastolic BP (mmHg)	81.2 ± 16.1	71.2 ± 19.1	0.26
Heart rate (/min)	80.8 ± 21.2	67.2 ± 17.1	0.12
Pain to balloon time (min)	225 ± 161	138 ± 51	0.01
LAD infarct	52/72 (72.2%)	6/6 (100%)	0.33
Flow-limiting bystander disease	8/72 (11.1%)	2/6 (33.3%)	0.35
Cardiac MRI			
Ejection fraction (%)	46 ± 13	39 ± 4	0.13
LGE (%)	32 ± 13	38 ± 16	0.53
Oedema (%)	44 ± 13	47 ± 18	0.78
Venous NPY concentration (pg/mL)	17.8 [10.6–30.2]	31.9 [27.8–47.7]	0.03

Values are mean ± standard deviation, *n* (%), median [interquartile range].

ACE, angiotensin converting enzyme; ATR, angiotensin receptor; BP, blood pressure; LAD, left anterior descending; LGE, late gadolinium enhancement; MI, myocardial infarction; MRI, magnetic resonance imaging; STEMI, ST-elevation myocardial infarction.

**Figure 1:**
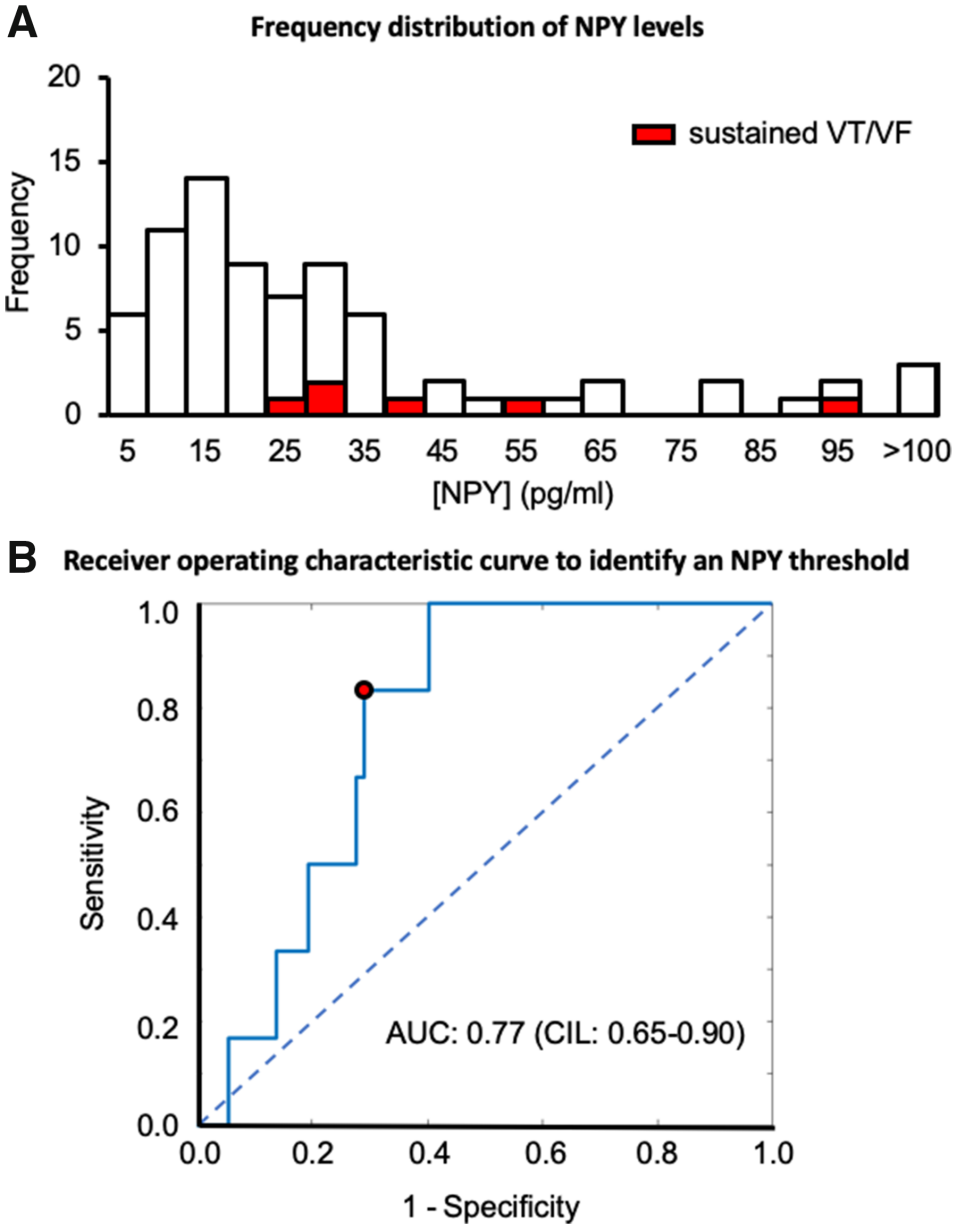
Venous neuropeptide Y levels identify patients with sustained ventricular tachycardia/ventricular fibrillation. (*A*) Frequency distribution of venous neuropeptide Y levels (pg/mL) across the study population (*n* = 78). Those patients experiencing sustained ventricular tachycardia or ventricular fibrillation are identified in red. (*B*) Receiver operating characteristic curve to find a venous neuropeptide Y level that best identifies patients with sustained ventricular tachycardia or ventricular fibrillation. The area under the curve (AUC) is 0.77 and the red point represents an neuropeptide Y level of 27.3 pg/mL which has a sensitivity of 0.83 and a specificity of 0.71.

The NPY levels of patients experiencing sustained VT/VF in relation to the entire NPY distribution are illustrated in *Figure 1A*. A receiver operating characteristic curve was used to define an NPY threshold that best identifies those patients with sustained VT/VF as shown in *Figure 1B*. A threshold NPY level of 27.3 pg/mL has a sensitivity of 0.83 and a specificity of 0.71 (area under the receiver operating characteristic curve: 0.77, 95% confidence intervals 0.65–0.90). The baseline characteristics of STEMI patients according to this threshold level are summarized in *Table 3*. Patients with high venous NPY levels were on average slightly older and a higher proportion were hypertensive. Usage of beta-blockers, angiotensin converting enzyme (ACE) inhibitors, and statins prior to presentation were similar in the two groups and both groups had similar pain to balloon times, left anterior descending artery infarctions, flow-limiting bystander disease, and haemodynamic parameters. There was no association between beta-blocker dose on admission (equivalent bisoprolol dose) and NPY levels (*r* = 0.06, *P* = 0.59). There were no significant differences in ejection fraction or infarct size as assessed by LGE between patients with low and high venous NPY levels above as shown in *Table 3*. The occurrence of all ventricular arrhythmias according to NPY levels are summarized in *Table 4*. Patients with high venous NPY levels had similar coupling intervals of ventricular ectopics compared to those with low NPY levels (474 ± 59 vs. 507 ± 33 ms, *P* = 0.24).

**Figure 2:**
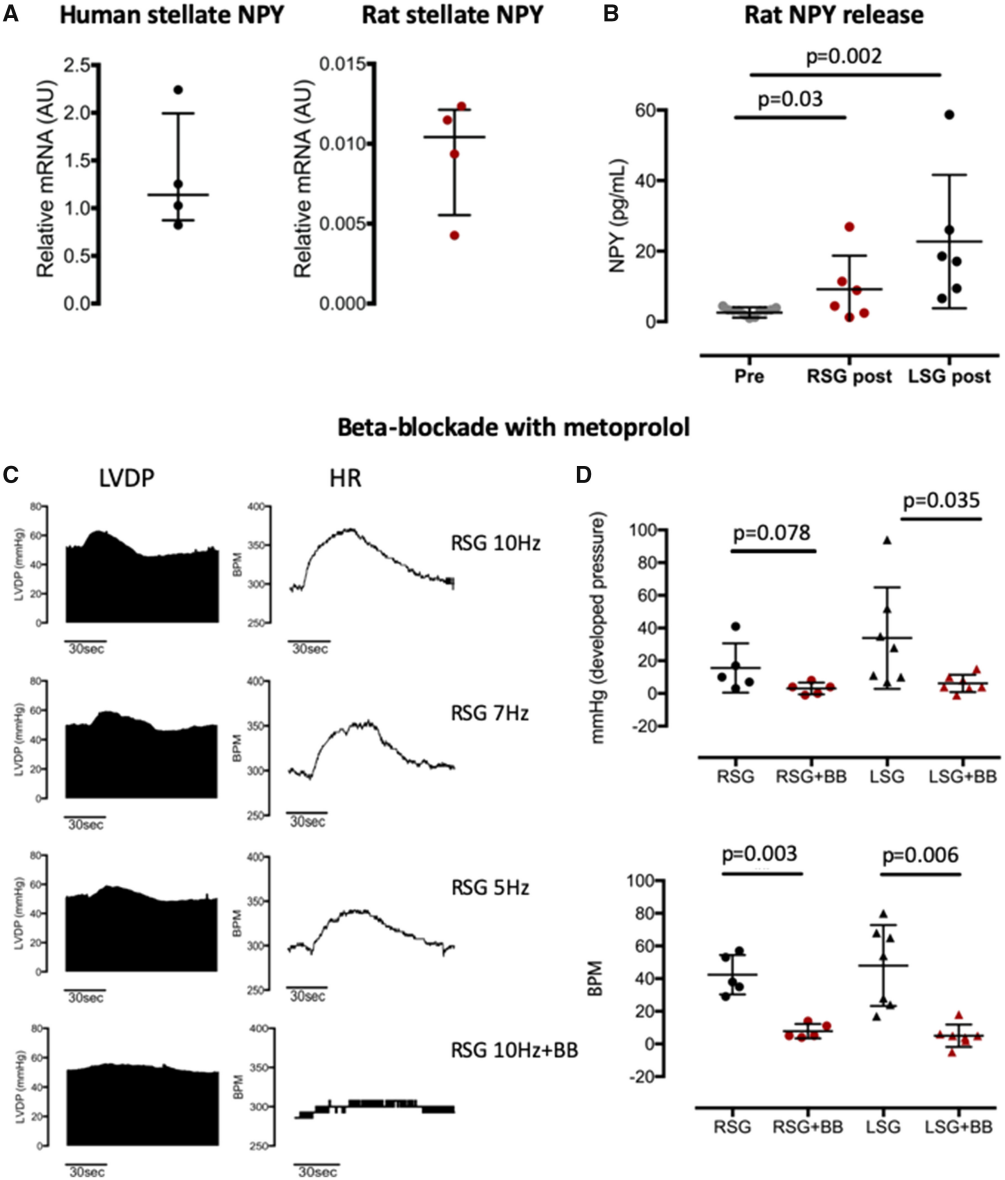
Stellate ganglia neuropeptide Y release and beta-blockade during sympathetic stimulation. (*A*) Neuropeptide Y mRNA is present in both human (*n* = 4) and rat (*n* = 4) stellate ganglia as identified using RT-qPCR. (*B*) Prolonged high frequency stimulation (10 Hz, 2 min) of right stellate ganglia (*n* = 6) or left stellate ganglia (*n* = 6) causes the release of neuropeptide Y into the perfusate of the isolated Langendorff perfused rat heart. (*C*) Example raw date trace showing the frequency-dependent increase in heart rate in beats per minute and left ventricular developed pressure (mmHg) in response to stellate stimulation in the isolated rat heart. (*D*) Beta-blockade with metoprolol (10 μmol/L) prevents the inotropic and chronotropic responses to either right stellate ganglia (*n* = 5) or left stellate ganglia (*n* = 7) stimulation at 10 Hz.

**Table 3:** Clinical characteristics according to venous NPY threshold

STEMI patients	Low NPY (*n* = 52)	High NPY (*n* = 26)	*P*-value
Age	60.6 ± 12.7	67.9 ± 10.4	0.01
Males	42/52 (80.8%)	19/26 (73.1%)	0.62
Cardiovascular risk factors			
Hypertension	19/52 (36.5%)	17/26 (66.4%)	0.03
Hyperlipidaemia	17/52 (32.7%)	15/26 (57.7%)	0.06
Diabetes mellitus	5/52 (9.6%)	5/26 (19.2%)	0.29
Current/Ex-smoker	42/52 (80.8%)	19/26 (73.1%)	0.62
Family history	16/52 (30.8%)	10/26 (38.5%)	0.67
Previous MI	3/52 (5.8%)	4/26 (15.4%)	0.21
Medications on admission			
Beta-blockers	7/52 (13.5%)	5/26 (19.2%)	0.74
ACE inhibitor/ATR antagonist	9/52 (17.3%)	9/26 (34.6%)	0.15
Statin	10/52 (19.2%)	8/26 (30.8%)	0.39
BP and heart rate at presentation			
Systolic BP (mmHg)	133.8 ± 24.5	135.8 ± 27.2	0.75
Diastolic BP (mmHg)	82.1 ± 15.7	77.2 ± 18.6	0.25
Heart rate (/min)	80.4 ± 20.9	78.4 ± 22.0	0.71
Pain to balloon time (min)	233 ± 162	189 ± 129	0.21
LAD infarct	37/52 (71.2%)	21/26 (80.8%)	0.52
Flow-limiting bystander disease	5/52 (9.6%)	5/26 (19.2%)	0.40
Cardiac MRI			
Ejection fraction (%)	47 ± 9	43 ± 10	0.14
LGE (%)	32 ± 14	32 ± 19	0.95
Oedema (%)	44 ± 16	45 ± 15	0.83
Venous NPY concentration (pg/mL)	13.9 [9.2–18.8]	45.2 [31.7–78.9]	<0.0001

Values are mean ± standard deviation, *n* (%), median [interquartile range].

ACE, angiotensin converting enzyme; ATR, angiotensin receptor; BP, blood pressure; LAD, left anterior descending; LGE, late gadolinium enhancement; MI, myocardial infarction; MRI, magnetic resonance imaging; STEMI, ST-elevation myocardial infarction.

**Table 4:** Ventricular arrhythmias according to venous NPY threshold

	STEMI arrhythmia frequency	
Arrhythmia	Low NPY	High NPY
None	35/52 (67.3%)	12/26 (46.2%)
VEs	11/52 (21.2%)	8/26 (30.8%)
NSVT	5/52 (9.6%)	1/26 (3.8%)
VT	1/52 (1.9%)	3/26 (11.5%)
VF	0	2/26 (7.7%)

NSVT, non-sustained ventricular tachycardia; STEMI, ST-elevation myocardial infarction; VEs, ventricular ectopic beats; VF, ventricular fibrillation; VT, sustained ventricular tachycardia.

*P* = 0.01 for VT/VF between experimental groups.

### Neuropeptide Y release and effects of ventricular electrophysiology and calcium handling

Using quantitiative reverse transcription polymerase chain reaction (RT-qPCR) NPY mRNA was identified in human (*n* = 4) and rat (*n* = 4) stellate ganglia tissue (*Figure 2A*). Prolonged (2 min) high frequency (10 Hz) stimulation of either left or right stellate ganglia in the integrated sympathetic nerve rat heart model resulted in the release of NPY in the coronary perfusate (*Figure 2B*), although release of NPY was greater with left stellate stimulation (*n* = 6) compared to right (*n* = 6). Stimulation of either stellate ganglia also resulted in a frequency-dependent increase in heart rate and left ventricular developed pressure, greater with left (*n* = 5) compared to right (*n* = 7) stellate stimulation, which could be abolished with the beta-blocker metoprolol (10 μmol/L, *Figure 2C *and* D*) even during prolonged (2 min) high frequency (10 Hz) stimulation (right stellate: 261 ± 16 b.p.m. to 269 ± 16 b.p.m., 51 ± 8 mmHg to 56 ± 11 mmHg, left stellate: 252 ± 25 b.p.m. to 257 ± 25 b.p.m., 65 ± 16 mmHg to 71 ± 18 mmHg).

**Figure 3:**
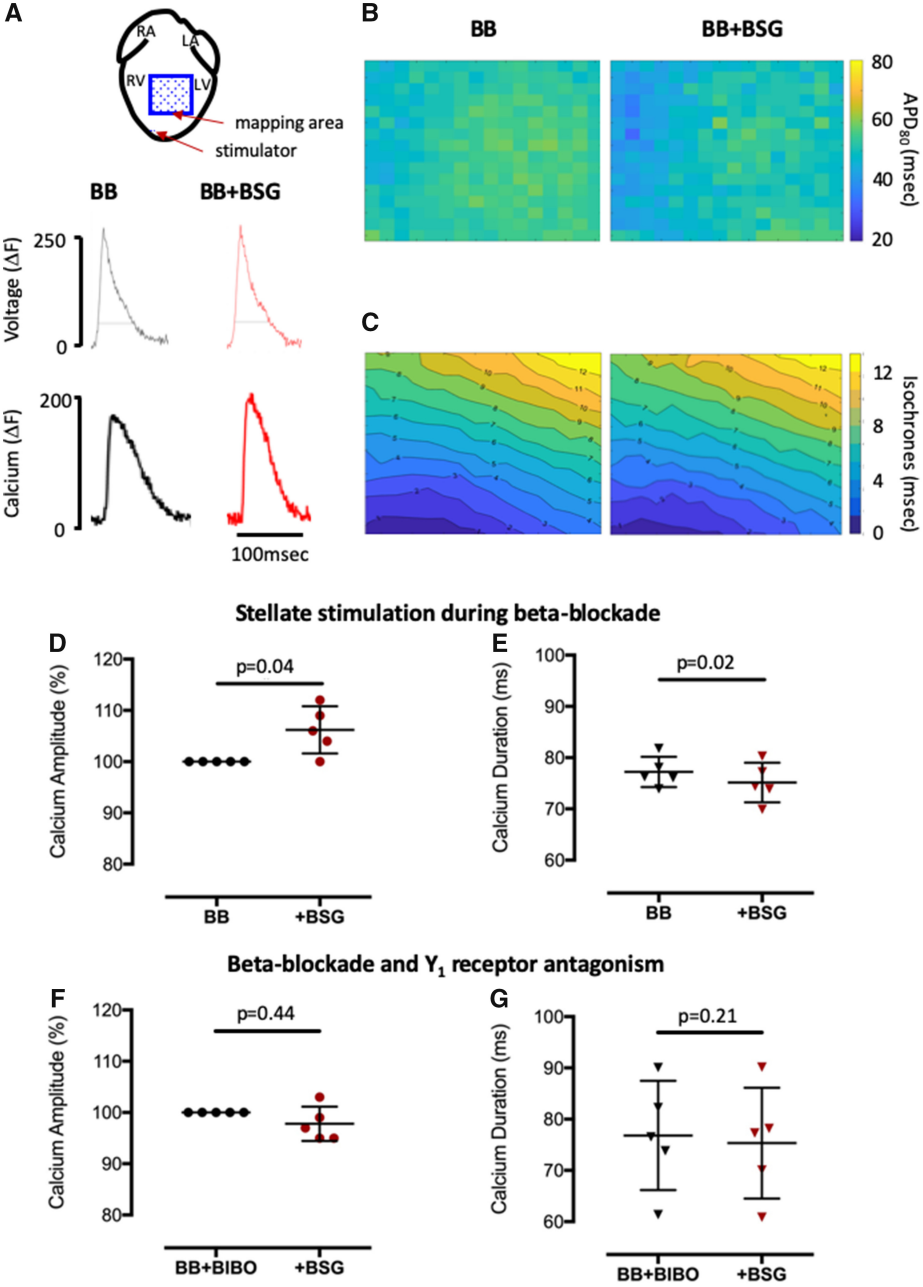
The effects of neuropeptide Y on ventricular calcium handling and voltage. (*A*) Schematic diagram showing the area of optical mapping and raw data trace showing the effects of prolonged high frequency (10 Hz, 2 min) bilateral stellate ganglia stimulation on both calcium and voltage. (*B* and *C*) Group mean data showing the effects of bilateral stellate ganglia in the presence of beta-blockade (*n* = 5) and (*D* and *E*), in the presence of beta-blockade and Y_1_ receptor antagonism with BIBO3304 (1 μmol/L, *n* = 5).

Optical mapping of voltage and intracellular calcium transients (using RH237 and Rhod2) at the anterior ventricular wall demonstrated that independent of heart rate (pacing at a cycle length of 140 ms), prolonged high-frequency stellate stimulation in the presence of metoprolol did not significantly change action potential duration (APD, 83 ± 21% *n* = 5) or activation time as shown in *Figure 3A–C*. However, there was a significant increase in the amplitude of the intracellular calcium transient accompanied by a significant shortening of the calcium transient duration as demonstrated in *Figure 3D *and* E*. The increase in calcium transient amplitude and shortening of the calcium transient duration could be prevented by a NPY Y_1_ receptor antagonist BIBO3304 (1 μmol/L, *n* = 5, *Figure 3F *and* G*).

### Neuropeptide Y and ventricular fibrillation threshold in the isolated heart

**Figure 4:**
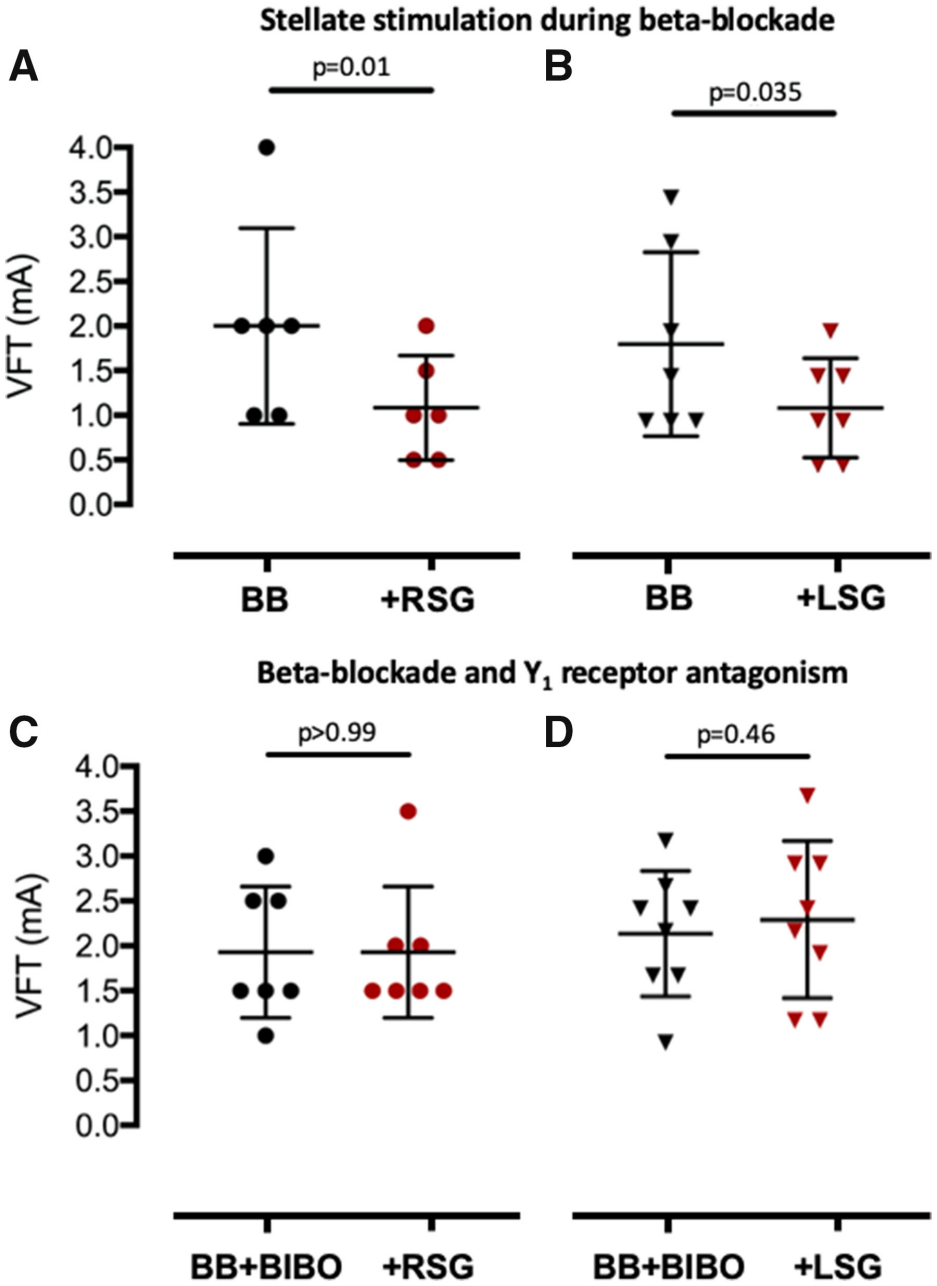
The effects of neuropeptide Y on ventricular fibrillation threshold. (*A* and *B*) The effects of prolonged high frequency (10 Hz, 2 min) right stellate ganglia (*n* = 6) or left stellate ganglia (*n* = 7) stimulation in the presence of beta-blockade with metoprolol (10 μmol/L) on ventricular fibrillation threshold assessed by burst pacing. (*C* and *D*) Group mean data showing the effects of right stellate ganglia (*n* = 7) or left stellate ganglia (*n* = 8) stimulation in the presence of beta-blockade and Y_1_ receptor antagonism with BIBO3304 (1 μmol/L).

To determine whether the increase in the magnitude and shortening in duration of the calcium transient predisposed the heart to ventricular arrhythmias, we measured ventricular fibrillation threshold (VFT) in response to burst pacing before and after prolonged high-frequency stellate ganglia stimulation in the presence of metoprolol. Right (*n* = 6) or left (*n* = 7) stellate stimulation in the presence of beta-blockade remained pro-arrhythmic with a significant reduction in VFT (*Figure 4A *and* B*) and this could be prevented by the addition of the Y_1_ receptor antagonist BIBO3304 (1 μmol/L, right *n* = 7, left *n* = 8) in combination with metoprolol (*Figure 4C *and* D*). This suggests that the pro-arrhythmic effect seen following prolonged high-frequency stellate ganglia stimulation in the presence of beta-blockade was due to NPY.

**Figure 5:**
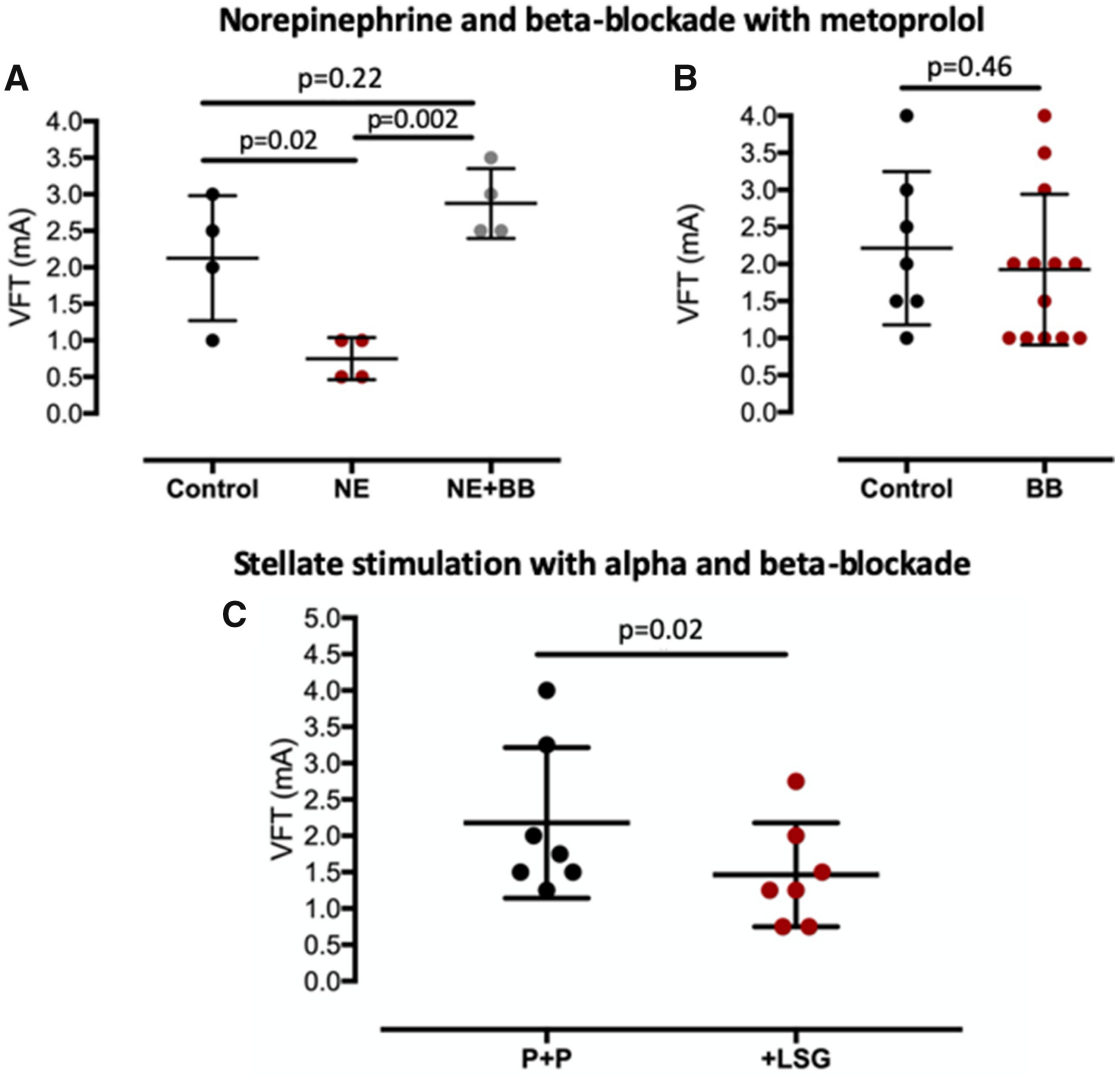
Effective adrenergic receptor blockade. (*A*) Beta-blockade with metoprolol (10 μmol/L) reverses the fall in ventricular fibrillation threshold in response to a maximal dose of norepinephrine (1 μmol/L, *n* = 4). (*B*) Beta-blockade does not influence ventricular fibrillation threshold compared to control (*n* = 7). (*C*) Combined beta and alpha-blockade (with propranolol, 1 μmol/L, and phentolamine, 1 μmol/L, P+P) does not prevent the fall in ventricular fibrillation threshold in response to prolonged high frequency (10 Hz, 2 min) left stellate ganglia stimulation (*n* = 7).

To further validate effective blockade of adrenergic receptor signalling, we demonstrated that metoprolol abolished the inotropic (control 70 ± 6 mmHg, norepinephrine 121 ± 18 mmHg, metoprolol and norepinephrine 63±16 mmHg) and chronotropic response (control 247±18 b.p.m., norepinephrine 296±5 b.p.m., metoprolol and norepinephrine 246±18 b.p.m.) to a maximal dose of norepinephrine (1 μmol/L) and also reversed a fall in VFT (*n* = 4) as shown in *Figure 5A*. Furthermore, metoprolol alone did not have a direct effect on VFT compared to control (*n* = 7) (*Figure 5B*). We also demonstrated that combined beta and alpha-receptor blockade (with propranolol, 1 μmol/L, and phentolamine, 1 μmol/L) also did not prevent the reduction in VFT to prolonged high-frequency stellate ganglia stimulation (*n* = 7) as shown in *Figure 5C*.

### Neuropeptide Y and experimental induced ST-elevation ischaemia reperfusion arrhythmias

To directly assess whether NPY triggers ventricular arrhythmia, we assessed the incidence and severity of arrhythmia in the rat in the setting of ST-elevation ischaemia reperfusion. NPY (*n* = 10) significantly increased the incidence of sustained VT and VF (60% vs. 10%) compared to control (*n* = 10) and this could be abolished with the Y_1_ receptor antagonists BIBO3304 (*n* = 10) as shown in *Table 5*.

**Table 5:** NPY and ventricular reperfusion arrhythmias following experimental ST-elevation ischaemia

		Reperfusion arrhythmia frequency		
ECG example	Arrhythmia	Control	NPY	NPY+BIBO
	None	8/10	1/10	1/10
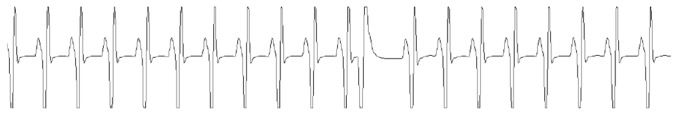	VEs	1/10	3/10	9/10
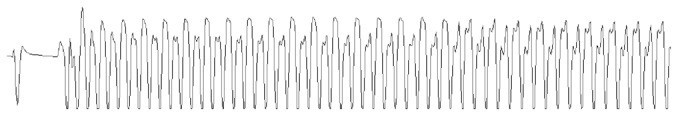	VT	1/10	1/10	0
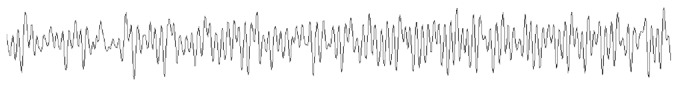	VF	0	5/10	0

*P* = 0.006 for VT/VF between experimental groups. ECG, electrocardiogram.

## Discussion

We report a novel mechanism by which sympathetic stimulation exerts a pro-arrhythmic effect on the ventricle. In the isolated heart, prolonged high frequency stimulation of the stellate ganglia releases NPY which acts via the Y_1_ receptor to increase the amplitude and shorten the duration of the ventricular myocyte calcium transient and lower ventricular fibrillation threshold, even in the presence of maximal beta-blockade (see *Take home figure*). Importantly, combining beta-blockade with a Y_1_ receptor antagonist abolishes the pro-arrhythmic effect of stellate ganglia stimulation. In patients presenting with STEMI treated with PPCI, NPY levels are associated with an increased incidence of ventricular arrhythmia in the immediate post-infarct period independent of classical risk factors such as late presentation, larger infarct size, and prior beta-blocker usage. Moreover, NPY also increases the incidence of ventricular arrhythmias during experimental ST-elevation ischaemia reperfusion and this can also be prevented by a Y_1_ receptor antagonist.

**Take home figure:**
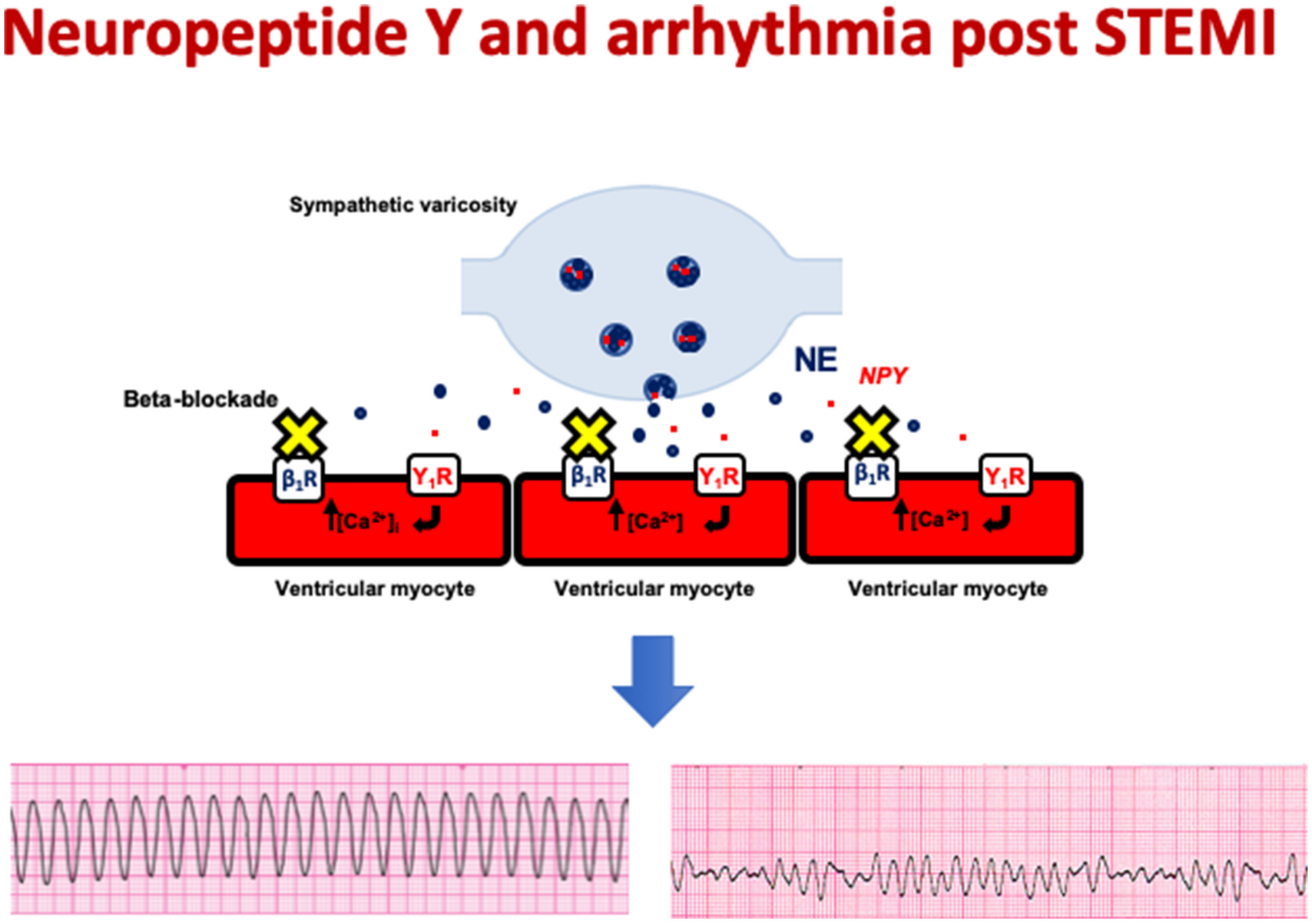
The sympathetic co-transmitter neuropeptide Y is released during ST-elevation myocardial infarction and via the Y_1_ receptor causes ventricular myocyte calcium overload and triggers life-threatening ventricular tachycardia and fibrillation.

Neuropeptide Y has a variety of effects on the cardiovascular system including acutely causing vasoconstriction,^8^ reducing acetylcholine release from the cardiac vagus and subsequent bradycardia,^15^ as well as promoting ventricular and vascular remodelling in the long term. It may also be involved in the pathogenesis of atherosclerosis.^10^ Here, we assess the acute effects of NPY release on ventricular electrophysiology, calcium handling, and arrhythmogenesis at the whole organ level and mitigate the effects of vasoconstriction by utilizing a constant flow system ensuring stable delivery of oxygen and metabolic substrate. Our isolated innervated heart preparation represents a useful model for the study of sympathetic control of arrhythmia. Other isolated heart models to date have employed spinal cord stimulation to produce efferent post-ganglionic sympathetic nerve stimulation although retrograde stimulation of afferent fibres, or activation of local reflexes may confound observations. Using our model, we clearly demonstrate that stimulation of the stellate ganglia leads to NPY release during prolonged high frequency stimulation and that whilst metoprolol was able to block the physiological effects of endogenous released and exogenous norepinephrine on heart rate and left ventricular developed pressure, it was unable to prevent an increase in the amplitude and shortening of duration of the intracellular calcium transient and reduction in VFT. This is suggestive of an additional substance being released during sympathetic stimulation that may have a pro-arrhythmic effect. Exogenous NPY can also act as an independent arrhythmic trigger during ST-elevation ischaemia reperfusion and, consistent with these observations, a combination of metoprolol and Y_1_ receptor blockade prevents the changes in intracellular calcium handling and decrease in VFT during stellate ganglia stimulation, or the triggered arrhythmias during ST-elevation ischaemia/reperfusion.

Although no animal model has identical electrophysiology and expresses the same combination of co-transmitters compared to human, the rat remains the best-studied model of NPY action at the single ventricular myocyte level in terms of electrophysiology and calcium handling, and NPY is the dominant sympathetic co-transmitter^16^ rather than galanin as seen in the mouse^17^ and guinea pig.^6^ Our data confirm NPY mRNA in both human and rat stellate ganglia which have also been demonstrated by immunohistochemistry in the former.^18^ Consistent with our findings in the whole heart, studies on isolated ventricular myocytes in the rat have demonstrated that NPY increases the magnitude of the intracellular calcium transient and causes diastolic calcium release events potentially due to coupling of NPY receptors to a phospholipase C, IP_3_ dependent pathway.^11^ Existing data on ion channel effects of NPY in isolated cardiomyocytes are variable. No alteration in sodium current (*I*_Na_) or delayed rectifier potassium current (*I*_k_) activity have been demonstrated^19^ but *I*_to_ was reduced.^12^ The effects on *I*_CaL_ have been shown to be species dependent with inhibition in guinea pig^13^ and stimulation in rat cardiac myocytes.^20^ However, we observe no overall effect of NPY on APD or activation times at fixed rate pacing in our model.

The pathophysiology of ventricular arrhythmias following ischaemia and reperfusion is complex. Ischaemia causes a reduction in cellular adenosine triphosphate (ATP), raises extracellular potassium through opening of ATP sensitive potassium channels, reduces inward rectifier potassium current (*I*_K1_) and the activity of the Na^+^/K^+^ ATPase. It also causes localized acidosis, uncoupling of gap junctions, and the generation of reactive oxygen species.^21^ There is an overall shortening of APD and refractory period as well as reduction in conduction velocity which predisposes to re-entrant arrhythmias. On reperfusion, acid extrusion via Na^+^/H^+^ exchange leads to a rise in intracellular Na^+^ and subsequently Ca^2+^ via NCX.^22^ Phosphorylation of calcium handling proteins by calcium-calmodulin dependent protein kinase II (CaMKII),^23^ and cellular calcium overload can lead to spontaneous sarcoplasmic reticulum calcium release, delayed afterdepolarization and initiate arrhythmias, whilst the spatial heterogeneity in repolarization, together with oedema and scar formation can also facilitate re-entry.^14^^,^^21^^,^^23^ All of these processes are exacerbated by sympathetic stimulation via the beta-adrenergic receptor, but even in the presence of beta-blockade, facilitation of sarcoplasmic reticulum calcium release by NPY signalling would be a potent pro-arrhythmic trigger.

The role of NPY as a non-adrenergic, sympathetic neuron derived pro-arrhythmic cotransmitter is supported by our observations in the OxAMI cohort of patients undergoing PPCI for STEMI. Direct stimulation of the cardiac sympathetic innervation leads to the appearance of NPY in coronary sinus (CS) blood,^24^ and we have shown that during STEMI CS and peripheral venous NPY levels are strongly correlated.^8^ Neuropeptide Y also has a long plasma half-life, and we have previously demonstrated that despite revascularization peripheral venous NPY levels remain significantly elevated for at least 48 h after treatment.^9^ In the OxAMI cohort, we therefore measured peripheral venous NPY levels immediately following PPCI and recorded ventricular arrhythmias during the first 48 h after reperfusion when 90% are known to occur. However, despite the lack of classical risk factors for ischaemia–reperfusion ventricular arrhythmias such as cardiogenic shock, late-presentation, larger infarct size, prior beta-blocker use, or longer pain to balloon time, patients with sustained VT/VF had significantly higher NPY levels. Interestingly, patients with high levels of NPY as defined by the receiver operating characteristic curve were more likely to be hypertensive, and recent data have also implicated NPY in the pathogenesis of hypertension.^25^

Historical studies undertaken in the 1980s before the advent of PPCI and modern medical treatment have shown that venous ‘NPY-like activity’ is elevated during ischaemic events and left ventricular failure and correlates with 1-year mortality.^26^^,^^27^ Unlike these early studies our assay is highly sensitive (2–3 pg/mL compared to >90 pg/mL) and selective (0% cross-reactivity with structurally similar peptides such as PYY, PP, GIP, ghrelin, proinsulin, or glucagon). We observe peripheral venous levels of NPY around three times that of patients undergoing elective coronary angiography with normal coronary arteries (7.8 pg/mL). Others have measured similarly low levels of venous NPY in healthy adults around 2 pg/mL.^28^ Using the same assay, we have also recently shown in patients with severe heart failure undergoing implantation of cardiac resynchronization therapy devices, that coronary sinus levels of NPY are significantly higher than in the STEMI population and are strongly associated with mortality.^29^

### Limitations

Ventricular arrhythmias causing out of hospital cardiac arrests have very low survival to discharge rates that are dependent on bystander recognition, adequate cardiopulmonary resuscitation, and early defibrillation.^30^ Such arrhythmias are clinically more serious than those witnessed peri-PPCI, although all sustained ventricular arrhythmias are potentially life-threatening. Sustained VT and VF occurring immediately pre- and post-PPCI are associated with higher rates of early and in-hospital mortality.^30^ It is challenging to thoroughly phenotype patients with out of hospital cardiac arrests prior to the arrest itself. These patients are often intubated prior to PPCI and therefore unable to consent, and frequently suffer from cardiogenic shock (an exclusion criteria in our study). Measuring NPY after such events is also complicated by the fact that the arrest itself will significantly raise sympathetic drive and NPY levels making it difficult to ascertain whether NPY played any role in their initiation. It would have been interesting to see whether the release profile of NPY throughout the 48 h of monitoring provides any additional prognostic information regarding late ventricular arrhythmia occurrence, but given that 90% of ventricular arrhythmias occur within the first 48 h,^30^ a far larger study would be required to address this question. We also do not have 12-lead electrocardiograms (ECGs) of all the observed arrhythmias in order to localize an electrocardiographic origin, as arrhythmias were mostly captured by standard ECG Holter monitoring and/or the rhythm strips from a defibrillator.

Whilst constant frequency stimulation is used universally for experimental neural stimulation, this clearly does not reproduce spontaneous neuronal firing behaviour observed *in vivo*. However, our stimulation parameters produce physiological changes in heart rate and left ventricular developed pressure and increased NPY levels as observed in the STEMI population. Inducing ventricular arrhythmias through pacing protocols could also be considered unphysiological, and the same criticism can also be applied to clinical VT stimulation testing (e.g. using the Wellens protocol). Ventricular fibrillation threshold testing in animal models allows for paired measurements of susceptibility before and after complex multi-step experimental interventions and therefore remains useful. However, we were careful to also demonstrate the effects of NPY on reperfusion arrhythmias induced by experimental ST-elevation ischaemia reperfusion in the rat as proof of principle that our findings are applicable to arrhythmogenesis following PPCI for STEMI.

### Clinical implications

Despite over half a century of research, the only primary prevention anti-arrhythmic drugs that reduce mortality following acute MI are beta-blockers. There is therefore an urgent need to identify new therapeutic targets and our data support the notion that blockers of the Y_1_ receptor may offer a novel pharmacological strategy in patients with ventricular arrhythmias, working synergistically with beta-blockers, and other secondary prevention medications. There is the possibility of administering such drugs via an intracoronary route at the time of PPCI, given their potential to also improve microvascular resistance and reduce infarct size,^8^ and/or via infusions during the first 48 h when NPY levels are at their highest,^9^ although the best approach is yet to be established.

It is also interesting to note that a variety of interventions aimed at reducing cardiac sympathetic drives such as cardiac sympathetic denervation, thoracic epidural anaesthesia, and renal denervation^2^^,^^3^ have shown promise in treating recurrent VT in patients with severe heart failure and long-QT syndrome, despite being treated with maximal doses of beta-blockers and receiving recurrent implantable cardioverter-defibrillator therapies. It may be that their efficacy is partly due to reducing the pro-arrhythmic action of NPY release in addition to simply reducing the release of catecholamines.

## Supplementary Material

ehz852_Supplementary_Table_1
