# Supplementary material for: The cardiac sympathetic co-transmitter neuropeptide Y is pro-arrhythmic following ST-elevation myocardial infarction despite beta-blockade
Source: Eur Heart J. 2019 Dec 13;41(23):2168–79. doi: 10.1093/eurheartj/ehz852 (PMC7299634; doi:10.1093/eurheartj/ehz852)
Supplement: ehz852_Supplementary_Table_1 [file ehz852_supplementary_table_1.docx]

**Supplementary Table 1. Details of patients experiencing sustained VT or VF following STEMI**

| **Age** | **Sex** | **Pain to balloon time (mins)** | **Venous NPY (pg/ml)** | **Rhythm**  **(cycle length)** | **Timing** | **Treatment** |
| --- | --- | --- | --- | --- | --- | --- |
| 86 | M | 175 | 23.3 | VT  (500msec) | 4 mins  post wire down | Spontaneous cardioversion |
| 54 | M | 93 | 27.3 | VT  (250msec) | 3 mins  post wire down | DC Cardioversion  (1 x 200J) |
| 55 | M | 87 | 29.1 | VT  (500msec) | 82 & 322 mins post wire down | Spontaneous cardioversion |
| 62 | M | 137 | 34.6 | VT  (440msec) | 5 & 130 mins post wire down | Spontaneous cardioversion |
| 49 | M | 218 | 52.1 | VF | 5 episodes  pre-PCI | Defibrillation to sinus with  1 x 200J each (+Amiodarone) |
| 76 | F | 120 | 90.8 | VF | 43 hours post wire down | Defibrillation to sinus with  1 x 150J |
